# Delivery room management of extremely low birth weight infants in Italian level III hospitals

**DOI:** 10.1186/1824-7288-41-S1-A44

**Published:** 2015-09-24

**Authors:** Daniele Trevisanuto, Irene Satariano, Nicoletta Doglioni, Giulio Criscoli, Francesco Cavallin, Camilla Gizzi, Claudio Martano, Fabrizio Ciralli, Flaminia Torielli, Paolo Ernesto Villani, Sandra Di Fabio, Lorenzo Quartulli, Luigi Giannini

**Affiliations:** 1 Children and Women's Health Department, Medical School University of Padua, Azienda Ospedaliera Padova, 35128 Padua, Italy; 2 Italian Army - Signals and Information Technology HQ - C4 Systems Integration Development, Treviso, Italy; 3 Independent Statistician, Padua, Italy; 4 Neonatal Intensive Care Unit Pediatric and Neonatal Department, “S. Giovanni Calibita” Fatebenefratelli Hospital - Isola Tiberina 00186 Rome, Italy; 5 Neonatal Intensive Care Unit Pediatric Department, Medical School University of Turin, Azienda Ospedaliera OIRM-S. Anna 10126 Torino, Italy; 6 Neonatal Intensive Care Unit Department of Mother and Infant Science, Fondazione IRCCS Ca’ Granda Ospedale Maggiore Policlinico, University of Milan 20122 Milan, Italy; 7 Neonatology Unit, University of Genova, Azienda Ospedaliera San Martino IRCCS – IST National Institute on Cancer Research 16100 Genova, Italy; 8 Neonatal Intensive Care Unit, Maternal and Pediatric Department, Carlo Poma Hospital, Mantova, Italy; 9 Neonatal Intensive Care Unit, Department of Mother and Infant Science, “San Salvatore” Hospital, L'Aquila, Italy; 10 Neonatology Unit, “A. Perrino” Hospital–ASL 72100 Brindisi, Italy; 11 Pediatric Department Medical School, University “La Sapienza” Rome, Azienda Ospedaliera Policlinico Umberto I, 00161 Rome, Italy

## Background

An increasing body of evidence suggests that delivery room management of extremely low birth weight (ELBW) infants may have a direct influence on their survival and long-term morbidity. 

We aimed evaluate the consistency of practice and the adherence to the International Guidelines in early delivery room management of ELBW infants in Italy. 

## Materials and methods

A questionnaire was sent to the directors of all Italian level III centers between April and August 2012.

## Results

There was a 92% (n=98/107) response rate. Participating centers reported an overall number of 198.322 births during 2011, and of these, 1933 were ELBW infants. Northern and Central centers had a higher median of births and of ELBW infants than Southern centers.

A provider skilled in neonatal resuscitation is present in high-risk deliveries in 46% of III level centers: this rate was higher in Northern (77.5%) than in Central (33.3%) and Southern (21.6%) centers. The team leader for neonatal resuscitation is generally a Pediatrician/Neonatologist (67.2%). The median delivery room temperature was 24°C (IQR: 22–25). Only 18 centers (20.2%) achieved a delivery room temperature over 25°C. A polyethylene bag/wrap was used by 54 centers (55.1%). Most centers had a pulse oxymeter (91/98, 92.9%) available in delivery room and used saturation targets (82/98, 83.7%). In Northern regions, one centre (2.5%) said it used oxygen concentrations >40% to initiate positive pressure ventilation in ELBW infants. These proportions were higher in the Central (14.3%) and Southern (16.2%) areas. A T-piece device for positive pressure ventilation was widely used (77/97, 79.4%). The percentage of ELBW infants intubated at birth had a median of 60% (IQR: 40%–80%), with the highest values in Central group (median 66%, IQR: 50%–75%). A median of 13% (IQR: 5%–30%) of ELBW infants received chest compressions at birth in Italy. 

## Conclusions

Overall, our results show a good adherence to the International Guidelines for Neonatal Resuscitation. Nevertheless, we found some relevant differences among the surveyed centers. Interventions to interpret and reduce the discrepancy among the different geographical areas are needed.
